# Examination of Skull Wounds by Physiological Methods: Practical Inferences for Professional Re-education

**Published:** 1919-01

**Authors:** 


### NEUROLOGY AND PSYCHIATRY

***Examination of Skull Wounds by Physiological Methods : Practical Inferences for Professional Re-education.*** By M. L. Binet. *Revue Interalliée pour les Mutilés de la Guerre*, June, 1918.

The wounds of the skull generally impel a certain psychic laziness; this may be determined mathematically by measuring the time elapsing between the production of a strong impression on the mind and the voluntary reaction to this. The electric chronometer of d'Arsonval may be used for this measure. It shows that reactions to visual impressions appear, in the case of normal subjects, after 19/100 of a second; to auditory and tactile impressions, after 14/100 of a second. These times were respectively: 23,2/100, 17,4/100, 15,5/100, in the case of a trephined soldier; and in the case of a shocked one: 41,6/100, 34,1/100, 35,5/100.

An exaggerated emotivity is often the consequence of a wound in the skull. This is controlled in the "Grand-Palais" in the following way: A strong emotion is produced by blank-firing a revolver in the neighborhood of the patient, and studying at the same time his breath, blood-circulation, and muscular trembling. All these show a long and intense reaction. For instance, the normal rate of the heart may reappear only after ten minutes.

Trephined men suffer very often from intense head-ache; any strain increases this and also the rate of the heart.

They often present motary disturbances marked by physiological trembling.

